# The Medical Profession and Secret Commissions

**Published:** 1910-07-23

**Authors:** 


### THE MEDICAL PROFESSION AND SECRET COMMISSIONS.

Members of the medical profession, whether individually or collectively, sometimes have to consider the question of secret commissions. For instance, in the working and management of a hospital, speculation with regard to such a matter may be rife, but actual proof of this particular form of underhand dealing is extremely hard to obtain. The transaction is well hidden from the light of day, as neither of the guilty parties wants it to be noised abroad. Cash need not pass, for "there are other ways of killing a cat besides drowning it in cream." Above all, scrupulous care is taken that no record of the transaction shall be made or preserved.

### How the Law Affects Hospitals.

The matter may come about in this way: Some new apparatus is required for the hospital. The governors naturally seek the advice of an expert—someone in whom they have confidence, who is to tell them where to go to get the best value for their money. He sends them the firm of A. B. & Co., who eventually secure the order and supply the goods. The work done may be fairly good; but it subsequently transpires that the expert has received a commission. Have his employers any remedy?

Until quite recently matters of this kind could not be inquired into except in courts of civil jurisdiction. To receive or to offer a bribe in the above circumstances was no criminal offence. Now, however, by virtue of the Prevention of Corruption Act, 1906, if any agent corruptly accepts or obtains, or agrees to accept or attempts to obtain, from any person, any gift or consideration as an inducement or reward for doing or forbearing to do any act in relation to his principal's affairs or business, or for showing or forbearing to show favour or disfavour to any person in relation to his principal's affairs or business; or if any person corruptly gives or agrees to give or offers any gift or consideration to any agent as an inducement or reward for doing or forbearing to do, or for having done or forborne to do any act in relation to his principal's affairs or business, or for showing or forbearing to show favour or disfavour to any person in relation to his principal's affairs or business; or if any person knowingly gives to any agent, or if any agent knowingly uses with intent to deceive his principal, any receipt, amount, or other document in respect of which the principal is interested, and contains any statement which is false or erroneous or defective, in any material particular, and which to his knowledge is intended to mislead the principal, he shall be guilty of a misdemeanour.

Applying this enactment to the case of the expert above mentioned he would clearly be guilty of a criminal offence. Further, the person who gave or even offered the commission to him would equally be guilty. It is worthy of notice, however, that, though this Act has been in force since January 1907, the law has hardly ever been put in motion. This is partly due to the fact that the fiat of the Attorney-General or of the Public Prosecutor has to be obtained before a prosecution is commenced; but undoubtedly the chief reason why people find it hopeless to institute proceedings is the difficulty of obtaining evidence.

### Civil Remedies.

Coming next to the civil remedies which are open to the victims of this kind of fraud, it may not be generally known that a secret commission may vitiate a whole transaction. The law is thus clearly stated in "Bowstead on Agency":—

"No agent is permitted to acquire any personal benefit in the course or by means of his agency without the knowledge and consent of the principal.

"Every agent must account to the principal for every benefit, and pay over to the principal every profit, acquired by him in the course or by means of the agency without such knowledge and consent, even if, in acquiring the benefit or profit, he incurred a risk of loss and the principal suffered no injury thereby."

### Presumption.

The law, therefore, presumes that if there has been a secret commission the employers have been injured by it and will not compel them to prove that injury. It has been laid down that where the transactions between a principal and his agent are severable, and in some of them he has been honest while in others he has been dishonest, he is entitled to his commission in all the instances in which he has been honest, but is not entitled to it in all the instances in which he has been dishonest. In a case which was heard in 1907 it was held that a house agent was not entitled to commission upon the sale of property upon the grounds that he had kept a material fact from the knowledge of his principal, and that he had made a secret profit. As Mr. Justice Lawrence said in that case: "A man who is employed to act for another as his agent is bound to exercise all the skill and all the knowledge he has of a particular business, all the diligence, all the zeal, and all the energy that he is capable of, and any interests he may have himself he is bound to exercise to the fullest extent for the sole and exclusive benefit of the person for whom he is acting."

### Commissions to General Practitioners.

This declaration of the law naturally leads us to the consideration of the question of secret commissions from a point of view which more nearly affects the medical profession itself. Suppose there are any dealings between the general practitioner and a so-called consultant, has the patient any remedy?

To put the matter quite plainly, suppose the consultant were to pay a commission to the general practitioner on every case which he sent to him, what would be the position? Such a transaction may not harm the patient much; but it would clearly amount to a breach of duty on the part of the general practitioner. His duty is to send his patient to the best man, and his discretion in the matter would clearly be biased by the receipt of a commission, which could clearly be recovered from him by the patient if he came to know of it. Apart from this, however, it is manifest that both general practitioner and consultant who entered into any such bargain would be guilty of "infamous conduct in a professional respect" which would bring them within the jurisdiction of the General Medical Council.

### Dichotomy.

Take another type of case. Suppose there was an understanding between a consultant and a general practitioner (Mr. A. B.) that if Mr. A. B. would send him patients the consultant would see all Mr. A. B.'s relations free of charge. Could such an arrangement be impeached? It is submitted that here, too, both consultant and general practitioner would be doing something which brings them within the grip of the criminal law, because the term "consideration" which is used in the Prevention of Corruption Act, 1906, includes "valuable consideration of any kind."

But perhaps we are setting up a bogey for the mere purpose of knocking it down. Because no cases of this pernicious practice (which has been dignified by the appellation "dichotomy") has hitherto come to light in this country we may probably assume that it does not exist. Further, with regard to the cases where there is an understanding that the consultant shall attend the relatives of the general practitioner free of charge, it is material to observe that every bargain in order to be criminal must be "corrupt," and the fact that it is usual and customary for medical practitioners to attend relations free of charge would be sufficient to disprove the existence of any corrupt motive.

